# Volker Heyse and Max Giger (eds): Erfolgreich in die Zukunft: Schlüsselkompetenzen in Gesundheitsberufen

**DOI:** 10.3205/zma000990

**Published:** 2015-11-16

**Authors:** Reinhard Putz

**Affiliations:** 1 Universität München, Anatomische Anstalt, München, Deutschland

## Recension

This book buys into the current trend of structuring every and any target-oriented interpersonal interaction in education, ongoing education and on-the-job training along competency boundaries. More than anything else, key competency has become a slogan that dominates every program and renders colorless any conventional educational approaches, which are oriented on knowledge, ability and ethos. This might be off-putting for many an experienced practitioner, but also for younger educators, since it means asking a series of foundational questions relevant to adult education, as well as challenging a number of established opinions.

This sizable volume gives voice to 51 authors through 29 individual contributions. It opens with an exhausive introduction, which ties together the independent chapters. This framework permits the reader to systematically approach relevant topics. There is a certain sense of mission discernible in the introduction, and it is defined by the editors' self-understanding, but it considerately and helpfully serves its purpose as a guide toward the concept and structure of the book.

The book itself covers five broad topics situated at the intersection points of multiple special themes and aspects of methodology:

- Developmental trends and supply models in the fields of education, ongoing education and on-the-job training
- Visions, new strategic approaches, examples of best practices
- Key leadership competencies
- Lateral collaboration among healthcare professionals
- Required competencies within the physician-therapist/caregiver-patient triad

The book does not lend itself to cover-to-cover reading. The individual contributions are too heterogenous for that. Rather, readers are encouraged to tentatively interact with a given topic and to be immersed in it, reflecting on their own interests and workplace challenges. The spectrum of the authors' personal opinions and experiences is wide, but without ever making a claim to completeness. Prolonged interaction with the texts will give rise to cross-connections, which at times lead to truly enlightening revelations. 

To experts with a practical background and who can draw on a wealth of information, this provides ample access to theoretical underpinnings and concepts that can be integrated into well-established and standardized training programs. On the other hand, theory-prone readers are given a plethora of practical examples that want to be applied.

The book's interdisciplinary approach deserves especial mention, as it memorably presents crossovers and parallels among various healthcare professions. This applies to the specific goals of different groups of professionals within healthcare, which at first sight appear discrepant, and to the way it achieves to present as inherently comparable many professional situations in which the ability to act as the situation demands — that is to say, the required competency – is interchangeable.

This is not a beginner's book. Its target audience consists primarily of experienced leaders in the healthcare professions. It is excellently suited as a foundational text for project planning and organization building. The wealth of information that is offered and the wide range of different approaches to definitions that is taken, as well as a large number of practical examples considered from many different angles, require a solid foundation and independent professional experience. For those readers who are willing to explore more-advanced connections beyond baseline facts and who are open to new ideas, this book is a treasure trove that they will want to have handy at their desk.

## Competing interests

The author declares, that he has no competing interests.

